# Study on securing care workers in Japan: Focusing on the management of older adult care workers

**DOI:** 10.1111/ggi.14682

**Published:** 2023-09-28

**Authors:** Kazuhiro Uchida

**Affiliations:** ^1^ Waseda University Tokyo Japan

Dear Editor,

Japan is projected to experience a shortage of 700 000 care workers by 2040, which poses a significant challenge.^1^ The proportion of care workers who are aged ≥60 years is steadily increasing annually. Currently, >20% of care workers are in this age group.^2^ Care workers require specialized knowledge and skills in nursing care, alongside physical strength, and dexterity to provide physical assistance and perform various tasks. Therefore, such roles should be appropriately assigned based on individual qualities, abilities or expertise. However, older adult care workers are often expected to carry out tasks similar to younger workers.

To secure an adequate number of care workers, it is important to establish an environment that allows older adult care workers to continue employment as they age. Therefore, exploratory interviews were carried out with human resource managers from eight older adult care facilities in Tokyo.

The objective of the present study was to examine current practices of managing older adult care workers. The interviews were carried out between February and March 2021, with a duration of ~60–90 min each. Interviews were recorded, transcribed and analyzed using a qualitative descriptive approach. The study was approved by the WASEDA University Research Ethics Committee, and all participants provided written consent.

The survey results yielded five categories and 22 subcategories. The first category, “Employment management,” included detailed explanations of the company's structure during recruitment interviews to ensure an understanding of older adult care workers. After recruitment, the suitability of older adult care workers in the workplace was evaluated based on input from on‐site employees. Flexible work schedules were introduced to accommodate individual convenience, considering factors, such as working hours and frequency. The work environments were aligned with older adult care workers' lifestyles, and work tasks assigned considering physical conditions to minimize burden. Adjustments to work schedules were made based on workplace conditions, such as coordinating work hours to match busy periods, or adjusting times for facility events. Initiatives were implemented to allow re‐entry after resignation based on lifestyle considerations, continuous proposal of work arrangements for sustained employment and age‐related employment restrictions were absent. Appropriate channels for consultation and opinion sharing were established, and adjustments made whenever issues arose.

The second category, “Ingenuity of the educational and operational support system,” encompassed practices, such as affixing name tags to seats during meal services to avoid mistakes, offering tailored support instructions, creating easily accessible and up‐to‐date reporting materials, and adapting to changes in visual and auditory capabilities, such as considering conditions for driving during certain times or adverse weather. Instead of merely teaching task sequences or routines, teaching methods were customized based on individual needs.

The third category, “Role clarification,” highlighted fostering connections with users based on older adult care workers' experienced perspective and empathy. Furthermore, older adult workers leverage their own experiences, conventional wisdom and knowledge gained from caring for their own parents, to guide younger generations.

The fourth category, “Evaluation systems,” involved regular acknowledgment and appreciation, setting self‐assessment objectives for minor accomplishments, and providing constructive feedback. Communication of the significance and purpose of working in caregiving, alongside valuing a proactive approach, and sincere dedication to new challenges and tasks, were emphasized.

The fifth category, “Fostering a workplace culture,” focused on promoting understanding of generational value differences, and discouraging the notion that younger generations should have the same physical abilities as older adults. Rather than segregating employees based on age groups, efforts were made to cultivate a multigenerational work culture that recognized the benefits of having a diverse team with varying perspectives and mutual stimulation.

In conclusion, the management of older adult care workers involves implementing “Employment management” tailored to their physical and lifestyle conditions, establishing personalized “Ingenuity of the educational and operational support system,” clarifying roles based on experience and age through “Role clarification,” creating inventive “Evaluation systems,” for work assessment, and “Fostering a workplace culture,” that seeks care workers' understanding and collaboration. Although there are numerous studies on employee management in older adult facilities, the present study focuses on older care workers. Based on the results, it is worth considering developing a management model for older adult workers that can be applied across caregiving facilities in the future.

## Disclosure statement

The author declares no conflict of interest.

## Data Availability

Data available on request from the authors.
